# Comparing stem cell mobilization with chemotherapy and cytokine (G-CSF) versus cytokine alone in myeloma patients (MOCCCA): a randomized phase II, open-label, non-inferiority trial

**DOI:** 10.1038/s41409-024-02468-z

**Published:** 2024-11-15

**Authors:** Barbara Jeker, Laura Thalmann, Ulrike Bacher, Henning Nilius, Gaëlle Rhyner, Martin Sökler, Susanne Soltermann, Annette Winkler, Corinne Vorburger, Michael Daskalakis, Michèle Hoffmann, Thomas Pabst

**Affiliations:** 1 Department of Medical Oncology, University Hospital and University of Bern, Bern, Switzerland; 2 Department of Hematology and Central Hematology Laboratory, University Hospital and University of Bern, Bern, Switzerland; 3 Department of Clinical Chemistry, Inselspital, University Hospital and University of Bern, Bern, Switzerland; 4 Department of Medical Oncology, Cantonal Hospital Fribourg, Fribourg, Switzerland; 5 Department of Medical Oncology and Hematology, Regional Hospital Thun, Thun, Switzerland; 6 Department of Medical Oncology and Hematology, Bürgerspital Solothurn, Solothurn, Switzerland; 7 Department of Medical Oncology and Hematology, Regional Hospital Biel, Biel, Switzerland

**Keywords:** Translational research, Myeloma

## Abstract

In fit patients with newly diagnosed myeloma, high-dose chemotherapy (HDCT) followed by autologous stem cell transplantation (ASCT) is considered standard of care. For mobilization of CD34+ cells for ASCT, combined cytotoxic chemotherapy and G-CSF is commonly used. However, the importance of cytostatic chemotherapy for reliable mobilization remains unclear. This prospective randomized phase II non-inferiority trial compared G-GSF only (G) compared to standard chemotherapy/G-CSF (CG) for CD34+ mobilization. The primary endpoint was a less than 15% difference in successful stem cell collection ( ≥ 5.0 × 10^6^ CD34+ cells/kg b.w. in a single day collection procedure without additional stimulation with plerixafor) with the G regimen. 136 patients were 1:1 randomized. With an 18% difference in favor of the CG therapy, the non-inferiority margin was not maintained (95% CI 1%, 34%, *p* = 0.04). The median total CD34+ yield was 9.99 × 10^6^/kg b.w. in CG patients and 7.42 × 10^6^/kg b.w. in patients with G-CSF alone (*p* < 0.001). Ultimately, 130 (96%) patients proceeded to HDCT with ASCT. There were no differences in adverse events, hematologic engraftment, quality of life, or pain perception between the groups. Our data indicate that G-CSF only is inferior to chemotherapy with G-CSF for peripheral CD34+ stem cell mobilization. Trial registration SNCTP #: SNCTP000002952; Trials.gov #: NCT03442673.

## Introduction

A rapidly growing number of novel emerging treatment options have recently been reported for relapsed/refractory myeloma patients, including CAR-T-cell therapies and bispecific antibodies. In first line treatment, a bortezomib and lenalidomide-based induction regimen eventually combined with anti-CD38 treatment followed by high dose chemotherapy (HDCT) with autologous stem cell transplantation (ASCT) remains standard of care in newly diagnosed and fit myeloma patients [1–8]. Despite several decades of experience with HDCT/ASCT in myeloma patients, and despite its frequent use, the optimal strategy for CD34+ mobilization preceding ASCT remains a matter of debate. Cyclophosphamide combined with granulocyte colony-stimulating factor (G-CSF) or G-CSF alone is most frequently used for peripheral stem cell mobilization [9, 10]. In Switzerland, the preferred mobilization treatment before ASCT is non-myelosuppressive chemotherapy with a single-dose of vinorelbine, a vinca alkaloid, combined with G-CSF, which provides reliable, safe and highly predictable collection of sufficient peripheral stem cells in a single-day procedure [11–14]. Vinorelbine is neurotoxic and can aggravate pre-existing neuropathy [15, 16]; therefore, in patients with pre-existing neuropathy gemcitabine, an antimetabolite, together with G-CSF is alternatively used and has been reported to be safe while somewhat less efficient for stem cell mobilization [13, 17]. However, up to 20% of patients are poorly mobilizing after both chemotherapy and G-CSF or with G-CSF alone. In such patients, the CXCR4 antagonist plerixafor is administered as additional rescue treatment usually enabling sufficient CD34+ mobilization [18–21].

Previous reports suggest that the use of G-CSF alone—as compared to chemotherapy plus G-CSF—is a reasonable mobilization strategy, even though stem cell yields are commonly lower [22–25]. However, it is currently unknown whether cytostatic chemotherapy is necessary at all for similarly reliable mobilization as with combined chemotherapy and G-CSF. This randomized, monocentric, non-blinded, phase II study aimed to demonstrate that the mobilization with G-CSF alone is non-inferior compared to a standard combined mobilization strategy with vinorelbine/gemcitabine and G-CSF. To our knowledge, it is the first randomized comparison between a non-myeloablative mobilization strategy and G-CSF only.

## Methods

### Study design

This was a randomized, monocentric, non-blinded, phase II trial recruiting from September 2018 to December 2022 at the University Hospital of Bern, Switzerland. The study had approval by the local Ethics Committee of Bern, Switzerland, with the decision number 2018-00615.

### Patients

Patients with multiple myeloma or amyloidosis had to be older than 18 years and considered clinically fit for subsequent consolidation with high-dose melphalan-based chemotherapy with autologous stem cell support. Eligible were patients after standard first-line induction treatment for multiple myeloma or second-line induction treatment in refractory multiple myeloma. Pregnancy had to be excluded in female patients. The data was collected by the study investigator in the RED Cap Software. All patients gave written informed consent.

### Randomization

In total, 136 patients were planned to be randomized 1:1 to either chemotherapy plus G-CSF (CG) or G-CSF only (G), using RED Cap software as randomization tool. Remission status at registration, preexisting peripheral neuropathy, and prior lenalidomide treatment were applied as parameters for a randomized stratification.

### Procedures

In the experimental arm (G) patients received G-CSF (filgrastim, weight adapted) twice daily from day 1 until the morning dose of the last day of stem cell collection. In the standard arm, (CG) patients received either a single dose of vinorelbine 35 mg/m^2^ intravenously over 10 min, or, in case of preexisting peripheral neuropathy, a single dose of gemcitabine 1250 mg/m^2^ intravenously over 30 min. on day 1, whereas G-CSF was applied twice daily from day 4 until the morning dose of the last day of stem cell collection.

In both groups, G-CSF was given at 60 Mio I.U. s.c./d (split in two daily doses of 30 Mio) for patients ≤69 kg body weight, at 78 Mio I.U. s.c./d (48 Mio I.U. in the morning and 30 Mio I.U. in the evening) for patients between 70 and 89 kg body weight, and at 96 Mio I.U. s.c./d (in two daily doses of 48 Mio I.U.) for patients ≥90 kg body weight.

The planned day for stem cell collection was day +5 in G patients, and day +8 in CG patients. The number of circulating peripheral CD34+ stem and progenitor cells was first assessed at the planned collection day and if applicable all subsequent days until stem cell apheresis. Apheresis procedure was initiated when at least 20.0 × 10^6^/l circulating CD34+ cells were present in the peripheral blood. At a CD34+ count of between 10.0 and 20.0 × 10^6^/l, stem cell collection was postponed to the following day and filgrastim was continued until collection. When the CD34+ count was below 10 × 10^6^/l, plerixafor was given for additional stimulation and the apheresis procedure was scheduled for the subsequent day.

CD34+ assessment in the peripheral blood and of stem cell harvests was performed by flow cytometry on a CANTO (BD Biosciences, Switzerland) flow cytometer according to ISHAGE guidelines.

Hematological engraftment was assessed daily and was defined as the first day of neutrophils rising above 0.5 × 10^9^/l and platelets rising above 20.0 × 10^9^/l for three days after HDCT in the absence of platelet transfusion.

The presence of del(17p), t(4;14) and t(14;16) were considered high risk cytogenetics.

### Outcomes

The primary objective was to demonstrate non-inferiority of mobilization with G-CSF alone compared to combined chemotherapy and G-CSF. Mobilization was considered successful if the total number of collected CD34+ cells in a single day procedure was exceeding 5.0 × 10^6^/kg body weight in the absence of additional use of plerixafor. This collection goal enables two ASCTs (or an additional CD34+ boost in the eventual case of engraftment failure). We assumed a success rate of 85%. The non-inferiority margin was 0.15 suggesting that G-CSF only was considered non-inferior to chemotherapy and G-CSF, if its success rate was no more than 15% worse than with CG.

The secondary objectives were to assess the need of additional use of plerixafor, pain and quality of life associated with the mobilization procedure in patients treated with chemotherapy and G-CSF as compared to cytokine stimulation alone. Additional secondary objectives were the assessment of differences in hematologic engraftment after ASCT and the comparison of adverse events between both treatment arms.

Toxicities and adverse events (AE) were assessed according to the CTCAE 5.0 during the study period (Supplementary Table S1). Quality of life and pain levels were assessed using the EORTC Q30 questionnaire filled at the first and the last day of the mobilization treatment [26, 27] (Supplementary Table S2). For pain assessment there was an additional numeric rating scale [28] (Supplementary Fig. S1).

### Statistical analyses

Sample size was calculated beforehand using the PASS11 software (NCSS Statistical Software, Kaysville, UT, United States). We set the alpha level to 0.05, the success rate to 0.85 and the non-inferiority margin to −15%. The sample size calculation revealed a minimal sample size of 136 patients (68 patients per arm) to achieve 80% power. For the primary endpoint, a non-inferiority margin of −15% was applied. In an intention-to-treat analysis, we calculated 95% confidence intervals for the difference in proportions between the two groups and applied a two-tailed chi-square test. Other categorical variables were presented as numbers and percentages. Statistical differences in these variables between the groups were assessed using either a chi-square test or, in cases where the patient count in one field of a 2 × 2 table was below 5, a Fisher’s exact test. For numerical variables, we presented the median and interquartile range. Differences between groups in numerical variables were examined using a Wilcoxon-Signed-Rank test. All analyses were done with R version 4.3.1 using the “stat” and “tableone” packages.

## Results

Between September 2018 and October 2022, we included 136 patients with multiple myeloma and amyloidosis at the University Hospital of Bern. In the CG arm, all patients received the stem cell mobilization treatment as planned: 33 (49%) patients received gemcitabine as mobilization chemotherapy, and 35 (51%) patients had vinorelbine. Two patients in the G arm did not receive the mobilization treatment, one patient due to an intermittent acute neurologic event (stroke) and one patient withdrew consent.

Patients and disease characteristics are summarized in Table 1. There were no statistically significant differences between both treatment groups. The median age in the cohort was 62.0 years (median IQR 55.8–67.0), 61.5 years (median IQR 56.8–66.3) in the CG arm and 63.0 years (median IQR 54.8–68.3) in the G arm. High-risk cytogenetics were observed in 15 (22%) patients in the CG group and in 11 (16%) patients in the G group. The most frequently used induction regimen was bortezomib, lenalidomide and dexamethasone (VRD) in both groups. 60 (88%) patients in the CG arm and 62 (91%) patients received ≤4 lenalidomide containing cycles (Table 2).

**Table 1:** Patient and disease characteristics.

Parameter	All patients	CG	G	*p*-value
Patients, *n* (%)	136 (100)	68 (100)	68 (100)	
Age at diagnosis, median (IQR)	62.00 (55.8–67.0)	61.50 (56.8–66.3)	63.00 (54.8–68.3)	0.26
Males/females, *n* (%)	79/57 (58/42)	44/24 (65/35)	35/33 (51/49)	0.16
R-ISS^a^, *n* (%)				0.51
I	33 (24)	15 (22)	18 (26)	
II	67 (49)	37 (54)	30 (44)	
III	23 (17)	12 (18)	11 (16)	
Type of Multiple Myeloma, *n* (%)				0.13
IgA	19 (14)	13 (19)	6 (9)	
IgD	1 (1)	1 (2)	0 (0)	
IgG	79 (58)	33 (49)	46 (68)	
IgM	1 (1)	1 (1)	0 (0)	
Light chain	36 (25)	20 (29)	16 (24)	
High risk cytogenetics, *n* (%)^a^	26 (19)	15 (22)	11 (16)	0.51
Amyloid deposition (bone marrow); *n* (%)^a^	6 (4)	1 (1)	5 (7)	0.16
Marrow infiltration^a^ median % (IQR)	60 (30–70)	60 (30–80)	50 (30–70)	0.48
Osteolytic Lesions^a^, *n* (%)	133 (98)	67 (99)	66 (97)	0.08
none	38 (28)	18 (26)	20 (30)	
single lesion	13 (10)	2 (3)	11 (16)	
2 lesions	7 (5)	4 (6)	3 (4)	
>2 lesions	54 (40)	32 (47)	22 (32)	
diffuse osteopenia	21 (15)	11 (16)	10 (15)	

Data are *n* (%) or median (IQR). High risk cytogenetics=del(17p), t(4;14), and t(14;16).

*CG* chemotherapy plus G-CSF group, *G* G-CSF only group, *R-ISS* revised multiple myeloma international staging system.

^a^Data not available for all randomised patients.

**Table 2:** Treatment prior to autologous stem cell transplantation.

Parameter	All patients	CG	G	*p*-value
Patients, *n* (%)	136 (100)	68 (100)	68 (100)	
1st induction regimen, *n* (%)				0.02
VRD	113 (83)	52 (77)	61 (90)	0.07
VCD	9 (7)	6 (9)	3 (4)	0.49
D-VRD	8 (6)	8 (12)	0 (0)	0.01
D-RD	3 (2)	0 (0)	3 (4)	0.24
D-VCD	2 (2)	1 (2)	1 (2)	1.00
RD	1 (1)	1 (2)	0 (0)	1.00
2nd induction regimen, *n* (%)	4 (3)	2 (3)	2 (3)	1.00
Lenalidomide, *n* (%)	126 (93)	62 (91)	64 (94)	0.74
≤4 cycles	122 (90)	60 (88)	62 (91)	0.78
>4 cycles	4 (3)	2 (3)	2 (3)	1.00
Daratumumab, *n* (%)	15 (11)	10 (15)	5 (7)	0.27
Bisphosphonates^a^, *n* (%)	83 (61)	47 (69)	36 (53)	0.14
Radiotherapy^a^, *n* (%)	40 (29)	24 (35)	16 (24)	0.21
RS prior to ASCT^a^, *n* (%)				0.52
SD	5 (4)	4 (6)	1 (1)	0.36
CR	33 (24)	15 (22)	18 (26)	0.69
VGPR	48 (35)	25 (37)	23 (34)	0.86
PR	48 (35)	23 (34)	25 (37)	0.86

Data are *n* (%) or median (IQR).

*CG* chemotherapy plus G-CSF group, *G* G-CSF only group, *VRD* Bortezomib, Lenalidomide, Dexamethasone, *VCD* Bortezomib, Cyclophosphamide, Dexamethasone, *D-VRD* Daratumumab, Bortezomib, Lenalidomide, Dexamethasone, *D-RD* Daratumumab, Lenalidomide, Dexamethasone, *D-VCD* Daratumumab, Bortezomib, Cyclophosphamide, Dexamethasone, *RD* Lenalidomide, Dexamethasone, *RS* remission status, *ASCT* autologous stem cell transplantation, *CR* complete remission, *VGPR* very good partial remission, *PR* partial remission, *SD* stable disease.

^a^Data not available for all randomised patients.

94 (69%) of all patients had successful stem cell mobilization with total collected CD34+ cells >5.0 × 10^6^ CD34+ cells/kg in a single day apheresis procedure without additional plerixafor, 53 (78%) patients in the CG arm and 41 (60%) in the G arm (*p* = 0.04) (Table 3). The difference between both arms equals 18% (95% CI: 1%, 34%) in favor of the CG therapy; thus, the non-inferiority margin (15%) was not maintained. Therefore, our data suggest that G-CSF monotherapy must be considered inferior to CG therapy regarding CD34+ cell collection.

**Table 3:** Details of CD34+ mobilization treatment and apheresis.

Parameter	All patients	CG	G	*p*-value
Patients, *n* (%)	136 (100)	68 (100)	68 (100)	
Leucocytes at start mobilization; ×10^9^/l leucocytes, median (IQR)	4.99 (4.33–5.89)	5.03 (4.42–5.87)	4.97 (4.06–5.91)	0.79
Platelets at start mobilization; ×10^9^/l platelets, median (IQR)	237 (183–310)	233.5 (173.5–308.75)	239 (191–310)	0.42
ASCC^a^, *n* (%)				
at planned collection date	96 (71)	48 (71)	48 (71)	0.93
in a single collection day	125 (92)	64 (94)	61 (89)	0.96
Total collected CD34+ cells; ×10^6^ CD34+ cells/kg b.w., median (IQR)	8.77 (6.45–12.18)	9.99 (7.40–13.88)	7.42 (5.61–10.56)	<0.001
Collected CD34+ cells in all apheresis days, *n* (%)				
>5.0 × 10^6^ CD34+ cells/kg b.w	118 (87)	62 (91)	56 (82)	0.26
>2.0; <5.0 × 10^6^ CD34+ cells/kg b.w.	14 (10)	5 (7)	9 (13)	0.27
<2.0 × 10^6^ CD34+ cells/kg b.w.	1 (1)	0 (0)	1 (1)	0.50
Collected CD34+ cells in a single day apheresis, *n* (%)				
>5.0 × 10^6^ CD34+ cells/kg b.w	113 (83)	60 (88)	53 (78)	0.23
>2.0; <5.0 × 10^6^ CD34+cells/kg b.w.	11 (8)	4 (6)	7 (10)	0.36
<2.0 × 10^6^ CD34+ cells/kg b.w.	1 (1)	0 (0)	1 (1)	0.49
>5.0 × 10^6^ CD34+ cells/kg b.w.in a single day apheresis, without plerixafor, *n* (%)	94 (69)	53 (78)	41 (60)	0.04
Plerixafor used, *n* (%)	27 (20)	9 (13)	18 (26)	0.06
single dose	24 (18)	8 (12)	16 (24)	
two doses	3 (2)	1 (2)	2 (3)	1.00

Data are *n* (%) or median (IQR).

*CG* chemotherapy plus G-CSF group, *G* G-CSF only group, *l* liter, *ASCC* autologous stem cell collection, *b.w* body weight.

^a^2 (2%) patients did not receive mobilization treatment.

**Fig. 1:**
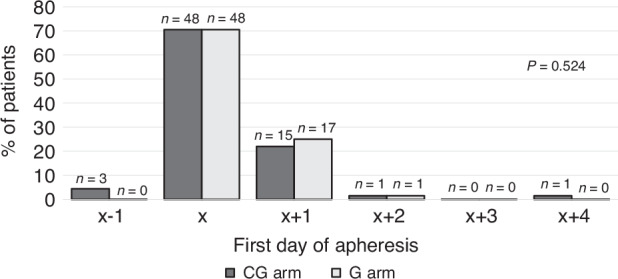
First day of CD34+ apheresis. CG chemotherapy plus G-CSF group, G G-CSF only group. X = planned apheresis day.

**Fig. 2:**
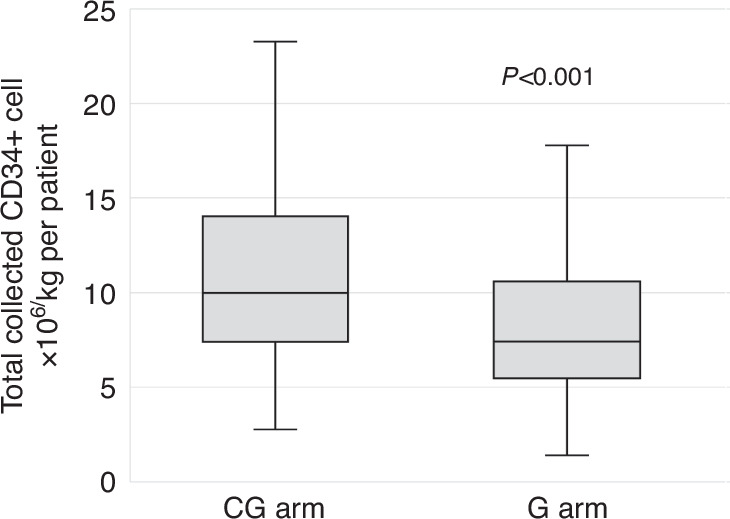
Total collected CD34+ cells. CG chemotherapy plus G-CSF group. G G-CSF only group. Median number of collected CD34+ cells, CG: 9.99 × 10^6^/kg b.w. (median IQR 7.40–13.88); G: 7.42 × 10^6^/kg b.w. (median IQR 5.61–10.56).

In both groups, stem cell collection was started at the planned apheresis day in 48 (71%) patients in each group (Fig. 1). A single day apheresis procedure was observed in 64 (94%) patients in the CG group and 61 (89%) patients in the G group. The median number of totally collected CD34+ cells was different in both groups, with 9.99 × 10^6^/kg b.w. (median IQR 7.40–13.88) in CG patients and 7.42 × 10^6^/kg b.w. (median IQR 5.61–10.56) in patients with G alone (*p* < 0.001) (Table 3, Fig. 2). In 62 (91%) patients in the CG arm, the mobilization goal ( ≥ 5.0 × 10^6^/kg b.w. CD34+ cells) was achieved, compared to 56 (82%) patients in the G arm (*p* = 0.26). There was a single patient with mobilization failure (CD34+ collected <2.0 × 10^6^/kg b.w.) in the G arm (who nevertheless proceeded to HDCT and ASCT with successful engraftment) (Table 3). More patients in the G arm needed additional plerixafor with 18 (26%) patients versus 9 (13%) patients in the CG arm, respectively (*p* = 0.06) (Table 3).

In total, 130 (96%) patients underwent high dose chemotherapy with melphalan and subsequent ASCT. 66 (97%) patients had ASCT in the CG arm, with two patients withdrawing consent. In the G arm, three patients withdrew consent (one of them before mobilization treatment started) and one patient had a stroke event before mobilization treatment, thus, 64 (94%) patients ultimately received ASCT.

The median time until neutrophil engraftment (neutrophil count > 0.5 × 10^9^/l) after ASCT was 11 (median IQR 10–15) days in CG patients compared to 12 (median IQR 11–17) days in G patients (*p* = 0.31), and the median time until platelet engraftment (platelet count ≥ 20 × 10^9^/l) was 12 days in both groups (*p* = 0.17) (Table 4). There was one single (1%) patient with an engraftment syndrome after HDCT and ASCT in G arm.

**Table 4:** Details of hospitalization and hematologic engraftment after high dose chemotherapy and autologous stem cell transplantation.

Parameter	All patients	CG	G	*p*-value
Patients, *n* (%)	136 (100)	68 (100)	68 (100)	
Patients with ASCT, *n* (%)	130 (96)	66 (97)	64 (94)	0.68
cell recovery in peripheral blood after ASCT in days; median (IQR)				
^a^Platelet count > 20 × 10^9^/l	12 (11–14)	12 (11–14)	12 (11–13)	0.17
^b^Platelet count >50 × 10^9^/l	17 (14–26)	16 (15–25)	18 (14–28)	0.76
^c^Platelet count >100 × 10^9^/l	29 (21–36)	30 (24–35)	29 (21–40)	0.93
^d^Neutrophil count >0.5 × 10^9^/l	12 (11–15)	11 (10–15)	12 (11–17)	0.32
^e^Neutrophil count >1.0 × 10^9^/l	13 (11–18)	13 (11–21)	13 (12–15)	0.48
^f^Leucocyte count >0.5 × 10^9^/l	11 (10–12)	11 (11–13)	11 (10–12)	0.60
^g^Leucocyte count >1.0 × 10^9^/l	12 (11–13)	12 (11–13)	12 (11–12)	0.67
Hospitalization days^h^, *n* (%)	21 (18–22)	20 (18–23)	21 (18–22)	0.92
Platelet transfusion^h^, *n* (%)	105 (77)	51 (79)	54 (86)	0.40
Units of platelets transfused per patient; median (IQR)	2 (1–3)	2 (1–3.5)	2 (1–3)	0.69
Red blood cell transfusion^h^, *n* (%)	43 (32)	21 (32)	22 (35)	0.90
Units of red blood cell transfused per patient; median (IQR)	1 (1–2)	2 (1–2)	1 (1–2)	0.12
Patients with fever^i^, *n* (%)	81 (60)	37 (54)	44 (65)	0.22
Pathogen identified	64 (47)	27 (40)	37 (54)	0.08
Bacterial infection	59 (43)	25 (37)	34 (50)	0.11
Viral infection	11 (8)	4 (6)	7 (10)	0.49
Fungal infection	0 (0)	0 (0)	0 (0)	1.00

Data are *n* (%) or median (IQR).

*CG* chemotherapy plus G-CSF group, *G* G-CSF only group, *ASCT* autologous stem cell transplantation.

Missing data or not reaching the specified number ^a^9 (7%); ^b^14 (10%); ^c^27 (20%); ^d^11 (8%); ^e^16 (12%); ^f^11 (8%); ^g^12 (9%); ^h^8 (6%); ^i^9 (7%).

There were no significant differences in the two groups for duration of hospitalization for HDCT and ASCT with a median of 20 days (median IQR, 18–23) in the CG group and 21 days (median IQR, 18–22) in the G group (*p* = 0.92). Most patients, 37 (54%) in the CG arm and 44 (65%) in the G arm, had at least one febrile episode during hospitalization for HDCT and ASCT (*p* = 0.22) (Table 4).

Adverse events from start of mobilization treatment until ASCT are summarized in the Supplementary Table 3 and indicate no significant difference between both arms.

**Fig. 3:**
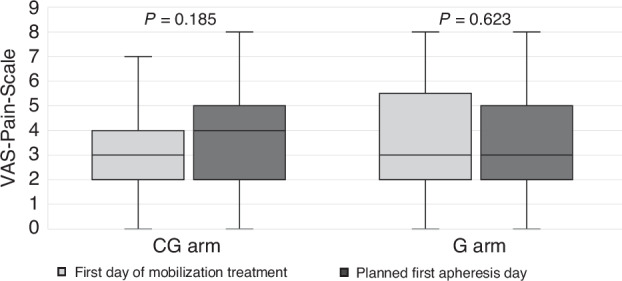
Pain perception during mobilization treatment. CG chemotherapy plus G-CSF group. G G-CSF only group, VAS visual analog scale (reaching from 1 to 10 points, 10 being the most painful ranking). Median VAS on first mobilization treatment day, CG: 3 (median IQR 2–4); G: 3 (median IQR 2–5.5). Median VAS on planned first apheresis day, CG: 4 (median IQR 2–5); G: 3 (median IQR 2–5).

Between both arms, there were no significant differences regarding the pain assessment reported on the first and on the last day of the mobilization treatment. The median pain level in the G arm was 3/10 at both time points (*p* = 0.31), whereas in the CG arm the median pain level was 3/10 on the first day of mobilization treatment and 4/10 on the collection day (*p* = 0.19) (Fig. 3). There was no difference in the quality of life assessment (EORTC Q30 questionnaire) under treatment between the two arms on the first and the last day of the mobilization procedure (Supplementary Table 4).

## Discussion

For fit myeloma patients being planned for HDCT/ASCT, chemotherapy such as with cyclophosphamide together with G-CSF or a strategy with G-CSF alone are commonly used for peripheral stem cell mobilization [9, 10, 29]. In Switzerland, the standard of care is a non-myeloablative chemotherapy regimen such as vinorelbine plus G-CSF or gemcitabine plus G-CSF providing low risk of fever in neutropenia, ambulatory cost-efficient management of the mobilization treatment and a reliable apheresis planning [11, 13, 15, 17]. Whether non-myeloablative chemotherapy is, in fact, needed for successful mobilization remains unclear, as a direct prospective comparison to a sole cytokine mobilization regimen has so far not been performed. In this prospective randomized phase-II study, we directly compared the mobilization potential of cytokine stimulation with G-CSF alone versus combined chemotherapy with vinorelbine and G-CSF (or in case of relevant neuropathy with gemcitabine and G-CSF) applying a non-inferiority design.

First, our study failed to demonstrate non-inferiority of G-CSF only with regards to peripheral CD34+ mobilization compared with combined non-myeloablative chemotherapy and G-CSF stimulation. In fact, the chemotherapy and G-CSF combination allowed to collect at least 5.0 × 10^6^ CD34+ cells/kg b.w. in the absence of plerixafor (as was the definition for a successful autologous CD34+ harvest in this study) in a single day procedure in 78% of all patients, whereas only 60% of patients reached this goal with G-CSF alone. Nevertheless, all but six patients (two in the CG arm and four in the G arm; all for unrelated reasons with patient wish in five cases, and a neurologic event before mobilization in one patient) ultimately proceeded to ASCT.

Our findings support previous data, comparing G-CSF and chemotherapy (cyclophosphamide) to G-CSF alone, indicating that collected stem cell yields are commonly higher in patients who received chemotherapy in addition to G-CSF, as the median number of collected CD34+ cells was 9.99 × 10^6^/kg b.w. in the chemotherapy group compared to 7.42 × 10^6^/kg b.w in patients with a sole cytokine mobilization (*p* < 0.001) [22, 23, 25]. With a G-CSF only mobilization regimen, >20% of patients are known to be poor CD34+ mobilizers [30]. In our population, 26% of patients in the G-CSF group received at least one dose of plerixafor. Even though the difference between our arms was not significant, plerixafor was given twice as often in the G-CSF only arm to achieve sufficient stem cell yields. Notably, with the option of salvage plerixafor and several collection days, all patients except one achieved a total number of collected CD34+ cells above 2.0 × 10^6^/kg b.w.

There were no significant differences in quality of life from start to end of mobilization treatment in both groups. The duration of hospitalization, number of febrile episodes and time to hematologic engraftment after HDCT showed no significant difference between the chemotherapy/G-CSF and the G-CSF only group.

Our data suggests no significant difference in adverse events during mobilization between the CG and the G arm. However, the report of patients with infections during or related to mobilization treatment with vinorelbine/gemcitabine is with 4 (6%) very low compared to the expected infection rate with an myeloablative mobilization treatment with cyclophosphamide which has been demonstrated to be 10–25% [22, 31, 32].

Our study suggests that G-CSF only is less effective than the chemotherapy/G-CSF combination with regards to CD34+ mobilization. This advocates that chemotherapy/G-CSF mobilization with vinorelbine/G-CSF for patients without pre-existing polyneuropathy or with gemcitabine/G-CSF for patients with pre-existing polyneuropathy should remain standard of care. However, the data also supports previous findings suggesting that—in cases where chemotherapy is preferred to be avoided for whatever reason—successful stem cell mobilization is also possible with G-CSF alone with the salvage option of additional plerixafor. However, we were not able to identify a clear subset of patients who would benefit from G-CSF only.

Even if the mobilization chemotherapy regimens with gemcitabine or vinorelbine are considered non-myeloablative and are usually well tolerated, cytotoxic agents may harbour the risk of fever in neutropenia, anaemia, gastrointestinal toxicities, and other complications. The selection of such regimens should consider these potential side effects.

Our study provides the first randomized comparison between a non-myeloablative mobilization strategy and G-CSF only, and these results may contribute to further define the most appropriate mobilization strategy for an individual myeloma patient.

## Supplementary information

Supplementary tables and figures

## Data Availability

The datasets generated during and analysed during the study are available from the corresponding author on reasonable request.

## References

[1] Attal M, Lauwers-Cances V, Hulin C, Leleu X, Caillot D, Escoffre M, et al. Lenalidomide, bortezomib, and dexamethasone with transplantation for myeloma. N. Engl J Med. 2017;376:1311–20. 28379796 10.1056/NEJMoa1611750 PMC6201242

[2] Dimopoulos MA, Moreau P, Terpos E, Mateos MV, Zweegman S, Cook G, et al. Multiple myeloma: EHA-ESMO clinical practice guidelines for diagnosis, treatment and follow-up†. Ann Oncol. 2021;32:309–22. 33549387 10.1016/j.annonc.2020.11.014

[3] Cavo M, Rajkumar SV, Palumbo A, Moreau P, Orlowski R, Bladé J, et al. International Myeloma Working Group consensus approach to the treatment of multiple myeloma patients who are candidates for autologous stem cell transplantation. Blood. 2011;117:6063–73. 21447828 10.1182/blood-2011-02-297325 PMC3293742

[4] Gillich C, Akhoundova D, Hayoz M, Aebi Y, Largiadèr CR, Seipel K, et al. Efficacy and safety of high-dose chemotherapy with treosulfan and melphalan in multiple myeloma. Cancers. 2023;15:2699. 37345036 10.3390/cancers15102699 PMC10216371

[5] Farag S, Bacher U, Jeker B, Legros M, Rhyner G, Lüthi JM, et al. Adding bendamustine to melphalan before ASCT improves CR rate in myeloma vs. melphalan alone: a randomized phase-2 trial. Bone Marrow Transpl. 2022;57:990–7. 10.1038/s41409-022-01681-y PMC9018972 35444232

[6] Bensinger WI. The role of hematopoietic stem cell transplantation in the treatment of multiple myeloma. J Natl Compr Cancer Netw. 2004;2:371–8. 10.6004/jnccn.2004.0030 19795599

[7] Voorhees PM, Kaufman JL, Laubach J, Sborov DW, Reeves B, Rodriguez C, et al. Daratumumab, lenalidomide, bortezomib, and dexamethasone for transplant-eligible newly diagnosed multiple myeloma: the GRIFFIN trial. Blood. 2020;136:936–45. 32325490 10.1182/blood.2020005288 PMC7441167

[8] Child JA, Morgan GJ, Davies FE, Owen RG, Bell SE, Hawkins K, et al. High-dose chemotherapy with hematopoietic stem-cell rescue for multiple myeloma. N. Engl J Med. 2003;348:1875–83. 12736280 10.1056/NEJMoa022340

[9] Awan F, Kochuparambil ST, Falconer DE, Cumpston A, Leadmon S, Watkins K, et al. Comparable efficacy and lower cost of PBSC mobilization with intermediate-dose cyclophosphamide and G-CSF compared with plerixafor and G-CSF in patients with multiple myeloma treated with novel therapies. Bone Marrow Transpl. 2013;48:1279–84. 10.1038/bmt.2013.52 23584435

[10] Chaudhary L, Awan F, Cumpston A, Leadmon S, Watkins K, Tse W, et al. Peripheral blood stem cell mobilization in multiple myeloma patients treat in the novel therapy‐era with plerixafor and G‐CSF has superior efficacy but significantly higher costs compared to mobilization with low‐dose cyclophosphamide and G‐CSF. J Clin Apher. 2013;28:359–67. 23765597 10.1002/jca.21280

[11] Samaras P, Pfrommer S, Seifert B, Petrausch U, Mischo A, Schmidt A, et al. Efficacy of vinorelbine plus granulocyte colony–stimulation factor for CD34+ hematopoietic progenitor cell mobilization in patients with multiple myeloma. Biol Blood Marrow Transplant. 2015;21:74–80. 25278456 10.1016/j.bbmt.2014.09.020

[12] Jeker B, Novak U, Mansouri Taleghani B, Baerlocher GM, Seipel K, Mueller BU, et al. NSAID treatment with meloxicam enhances peripheral stem cell mobilization in myeloma. Bone Marrow Transpl. 2018;53:175–9. 10.1038/bmt.2017.234 29058701

[13] Jeker B, Farag S, Taleghani BM, Novak U, Mueller BU, Li Q, et al. A randomized evaluation of vinorelbine versus gemcitabine chemotherapy mobilization of stem cells in myeloma patients. Bone Marrow Transpl. 2020;55:2047–51. 10.1038/s41409-020-0875-8 32214229

[14] Bühler S, Akhoundova D, Jeker B, Legros M, Seipel K, Daskalakis M, et al. Stem cell mobilization with Ixazomib and G-CSF in patients with multiple myeloma. Cancers. 2023;15:430. 36672379 10.3390/cancers15020430 PMC9856560

[15] Keller S, Seipel K, Novak U, Mueller BU, Taleghani BM, Leibundgut K, et al. Neurotoxicity of stem cell mobilization chemotherapy with vinorelbine in myeloma patients after bortezomib treatment. Leuk Res. 2015;39:786–92. 25891070 10.1016/j.leukres.2015.03.015

[16] Pace A, Bove L, Nistico C, Ranuzzi M, Innocenti P, Pietrangeli A, et al. Vinorelbine neurotoxicity: clinical and neurophysiological findings in 23 patients. J Neurol Neurosurg Psychiatry. 1996;61:409–11. 8890782 10.1136/jnnp.61.4.409 PMC486585

[17] Mueller BU, Keller S, Seipel K, Mansouri Taleghani B, Pabst T. Stem cell mobilization chemotherapy with gemcitabine is effective and safe in myeloma patients with bortezomib induced neurotoxicity. Blood. 2015;126:5436. 10.3109/10428194.2015.1079315 26294015

[18] DiPersio JF, Stadtmauer EA, Nademanee A, Micallef INM, Stiff PJ, Kaufman JL, et al. Plerixafor and G-CSF versus placebo and G-CSF to mobilize hematopoietic stem cells for autologous stem cell transplantation in patients with multiple myeloma. Blood. 2009;113:5720–6. 19363221 10.1182/blood-2008-08-174946

[19] Schmid A, Friess D, Mansouri Taleghani B, Keller P, Mueller BU, Baerlocher GM, et al. Role of plerixafor in autologous stem cell mobilization with vinorelbine chemotherapy and granulocyte-colony stimulating factor in patients with myeloma: a phase II study (PAV-trial). Leuk Lymphoma. 2015;56:608–14. 24884311 10.3109/10428194.2014.927454

[20] Bilgin YM. Use of plerixafor for stem cell mobilization in the setting of autologous and allogeneic stem cell transplantations: an update. J Blood Med. 2021 12:403–12. 34104027 10.2147/JBM.S307520 PMC8180285

[21] Maechler M, Bacher U, Daskalakis M, Nilius H, Nagler M, Taleghani BM, et al. Long‐term safety of the stem cell releasing compound plerixafor for peripheral stem cell collection in myeloma patients. Hematol Oncol. 2023;41:583–6. 35920140 10.1002/hon.3055

[22] Gertz MA, Kumar SK, Lacy MQ, Dispenzieri A, Hayman SR, Buadi FK, et al. Comparison of high-dose CY and growth factor with growth factor alone for mobilization of stem cells for transplantation in patients with multiple myeloma. Bone Marrow Transpl. 2009;43:619–25. 10.1038/bmt.2008.369 PMC2914501 18997825

[23] Chua CC, Lim HY, Chai KL, Ong J, Sim S, Wood C, et al. Peripheral blood stem cell mobilisation with G-CSF alone versus G-CSF and cyclophosphamide after bortezomib, cyclophosphamide and dexamethasone induction in multiple myeloma. Bone Marrow Transpl. 2018;53:1116–23. 10.1038/s41409-018-0152-2 29523889

[24] Bargetzi MJ, Passweg J, Baertschi E, Schoenenberger A, Gwerder C, Tichelli A, et al. Mobilization of peripheral blood progenitor cells with vinorelbine and granulocyte colony-stimulating factor in multiple myeloma patients is reliable and cost effective. Bone Marrow Transpl. 2003;31:99–103. 10.1038/sj.bmt.1703787 12621490

[25] Laszlo D, Marcacci GP, Martino M, Radice D, Rabascio C, Lucchetti B, et al. A comparison of chemo-free strategy with G-CSF plus plerixafor on demand versus intermediate-dose cyclophosphamide and G-CSF as PBSC mobilization in newly diagnosed multiple myeloma patients: an Italian explorative cost analysis. Transfus Apheresis Sci. 2020;59:102819. 10.1016/j.transci.2020.102819 32499108

[26] Proskorovsky I, Lewis P, Williams CD, Jordan K, Kyriakou C, Ishak J, et al. Mapping EORTC QLQ-C30 and QLQ-MY20 to EQ-5D in patients with multiple myeloma. Health Qual Life Outcomes. 2014;12:35. 24618388 10.1186/1477-7525-12-35 PMC4007827

[27] Kaasa S, Bjordal K, Aaronson N, Moum T, Wist E, Hagen S, et al. The EORTC core quality of life questionnaire (QLQ-C30): validity and reliability when analysed with patients treated with palliative radiotherapy. Eur J Cancer. 1995;31:2260–3. 10.1016/0959-8049(95)00296-0 8652253

[28] Boonstra AM, Stewart RE, Köke AJA, Oosterwijk RFA, Swaan JL, Schreurs KMG, et al. Cut-off points for mild, moderate, and severe pain on the numeric rating scale for pain in patients with chronic musculoskeletal pain: variability and influence of sex and catastrophizing. Front Psychol. 2016;7. 10.3389/fpsyg.2016.01466. 10.3389/fpsyg.2016.01466 PMC5043012 27746750

[29] International Myeloma Foundation. https://www.myeloma.org/autologous-stem-cell-transplant (accessed 12 Mar 2024).

[30] Li Y, Qiu X, Lei Y, Zhou R. G-CSF + plerixafor versus G-CSF alone mobilized hematopoietic stem cells in patients with multiple myeloma and lymphoma: a systematic review and meta-analysis. Ann Med. 2024;56:2329140. 38470973 10.1080/07853890.2024.2329140 PMC10939106

[31] Sarıcı A, Erkurt MA, Bahçecioğlu ÖF, Gök S, Kuku İ, Biçim S, et al. Filgrastim alone versus cyclophosphamide and filgrastim for mobilization in multiple myeloma patients. Transfus Apher Sci. 2021;60. 10.1016/j.transci.2021.103159. 10.1016/j.transci.2021.103159 34034961

[32] Johnsrud A, Ladha A, Muffly L, Shiraz P, Goldstein G, Osgood V, et al. Stem cell mobilization in multiple myeloma: comparing safety and efficacy of cyclophosphamide +/− plerixafor versus granulocyte colony-stimulating factor +/− plerixafor in the lenalidomide era. Transpl Cell Ther. 2021;27:590.e1–590.e8. 10.1016/j.jtct.2021.04.016 PMC8378276 33915323

