# Supplementary material for: Comparing stem cell mobilization with chemotherapy and cytokine (G-CSF) versus cytokine alone in myeloma patients (MOCCCA): a randomized phase II, open-label, non-inferiority trial
Source: Bone Marrow Transplant. 2024 Nov 15;60(3):270–6. doi: 10.1038/s41409-024-02468-z (PMC11893443; doi:10.1038/s41409-024-02468-z)

**Supplementary material: Comparing stem cell mobilization with chemotherapy and cytokine (G-CSF) versus cytokine alone in myeloma patients (MOCCCA-Trial):**

**A randomized phase II, open-label, non-inferiority trial**

**Table of contents**

Supplementary Table S1. CTC adverse events version 5.0 2

Supplementary Table S2. EORTC QLQ-C30 Version 3.04

Supplementary Table S3. Details of severe adverse events6

Supplementary Table S4. Quality of Life Assessment (EORTC Q30)7

Supplementary Figure S1. Numeric pain rating scale 8

Supplementary Table S1. CTC adverse events version 5.0

| **Adverse event/ toxicity grade** | **1 – mild** | **2 – moderate** | **3 – severe** | **4 – life threatening** |
| --- | --- | --- | --- | --- |
| **Neutropenia** | < LLN – 1500/mm³ | < 1500 – 1000/mm³ | < 1000 – 500/mm³ | < 500/mm³ |
| **Thrombocyto-penia** | < LLN – 75.000/mm³ | < 75.000 – 50.000/mm³ | < 50.000 – 25.000/mm³ | < 25.000/mm³ |
| **Anemia** | Hb < LLN –  10.0 g/dl | Hb 10.0 – 8.0 g/dl | Hb <8.0 g/dl | Life-threatening consequences; urgent intervention indicated |
| **Nausea** | Loss of appetite without alteration in eating habits | Oral intake decreased without significant weight loss, dehydration or malnutrition | Inadequate oral caloric or fluid intake; tube feeding, TPN, or hospitalization indicated | - |
| **Vomiting** | Intervention not indicated | Outpatient i.v. hydration; medical intervention indicated | Tube feeding, TPN, or hospitalization indicated | Life-threatening consequences |
| **Diarrhea** | Increase of <4 stools per day over baseline; mild increase in ostomy output compared to baseline | Increase of 4 - 6 stools per day over baseline; moderate increase in ostomy output compared to baseline; limiting instrumental ADL | Increase of >=7 stools per day over baseline; hospitalization indicated; severe increase in ostomy output compared to baseline; limiting self care ADL | Life-threatening consequences; urgent intervention indicated |
| **Fatigue** | Fatigue relieved by rest | Fatigue not relieved by rest; limiting instrumental ADL | Fatigue not relieved by rest, limiting self care ADL | - |
| **Mucositis oral** | Asymptomatic or mild symptoms; intervention not indicated | Moderate pain or ulcer that does not interfere with oral intake; modified diet indicated | Severe pain; interfering with oral intake | Life-threatening consequences; urgent intervention indicated |
| **Peripheral sensory neuropathy** | Asymptomatic | Moderate symptoms; limiting instrumental ADL | Severe symptoms; limiting self care ADL | Life-threatening consequences; urgent intervention indicated |
| **Peripheral motor neuropathy** | Asymptomatic; clinical or diagnostic observations only | Moderate symptoms; limiting instrumental ADL | Severe symptoms; limiting self care ADL | Life-threatening consequences; urgent intervention indicated |
| **Febrile Neutropenia** | - | - | ANC <1000/mm3 with a single temperature of >38.3°C or a sustained temperature of ≥38°C for more than one hour | Life-threatening consequences; urgent intervention indicated |
| **Infusion related reaction** | Mild transient reaction; infusion interruption not indicated; intervention not indicated | Therapy or infusion interruption indicated but responds promptly to symptomatic treatment (e.g., antihistamines, NSAID, narcotics, i.v. fluids); prophylactic medications indicated for <=24 hours | Prolonged (e.g., not rapidly responsive to symptomatic medication and/or brief interruption of infusion); recurrence of symptoms following initial improvement; hospitalization indicated for clinical sequelae | Life-threatening consequences; urgent intervention indicated |
| **Allergic reaction** | Systemic intervention not indicated | Oral intervention indicated | Bronchospasm; hospitalization indicated for clinical sequelae; intravenous intervention indicated | Life-threatening consequences; urgent intervention indicated |
| **Anaphylaxis** | - | - | Symptomatic bronchospasm, with or without urticaria; parenteral intervention indicated; allergy-related edema/angioedema; hypotension | Life-threatening consequences; urgent intervention indicated |
| **Bone pain / back pain** | Mild pain | Moderate pain; limiting instrumental ADL | Severe pain; limiting self care ADL |  |
| **Skelettal pain** | Asymptomatic or mild symptoms; intervention not indicated | Moderate; minimal, local or noninvasive intervention indicated; limiting age-appropriate instrumental ADL | Severe or medically significant but not immediately life-threatening; hospitalization or prolongation of existing hospitalization indicated; limiting self-care ADL |  |
| **Dysphagia** | Symptomatic, able to eat regular diet | Symptomatic and altered eating/ swallowing | Severely altered eating/swallowing; tube feeding, TPN, or hospitalization indicated | Life-threatening consequences; urgent intervention indicated |
| **Weight loss** | 5 to <10% from baseline; intervention not indicated | 10 - <20% from baseline; nutritional support indicated | >=20% from baseline; tube feeding or TPN indicated | - |
| **Thrush** | Asymptomatic; local symptomatic management | Oral intervention indicated (e.g., antifungal) | i.v. antifungal intervention indicated | - |

https://ctep.cancer.gov/protocoldevelopment/electronic_applications/ctc.htm#ctc_50

Supplementary Table S2. EORTC QLQ-C30 Version 3.0

UPN: …………………...  before mobilization (day 1)  after mobilization (day 5 or 8)

We are interested in some things about you and your health. Please answer all of the questions yourself by circling the number that best applies to you. There are no "right" or "wrong" answers. The information that you provide will remain strictly confidential.

|  |  | | | **Not**  **at all** | | **A**  **little** | **Quiet**  **a bit** | **Very**  **much** |
| --- | --- | --- | --- | --- | --- | --- | --- | --- |
| 1. | Do you have any trouble doing strenuous activities, like carrying a heavy shopping bag or a suitcase? | | | 1 | | 2 | 3 | 4 |
| 2. | Do you have any trouble taking a long walk? | | | 1 | | 2 | 3 | 4 |
| 3. | Do you have any trouble taking a short walk outside of the house? | | | 1 | | 2 | 3 | 4 |
| 4. | Do you need to stay in bed or a chair during the day? | | | 1 | | 2 | 3 | 4 |
| 5. | Do you need help with eating, dressing, washing yourself or using the toilet? | | | 1 | | 2 | 3 | 4 |
|  |  | | |  | |  |  |  |
|  | | **During the past week** | **Not**  **at all** | | **A**  **little** | | **Quiet**  **a bit** | **Very**  **much** |
| 6. | | Were you limited in doing either your work or other daily activities? | 1 | | 2 | | 3 | 4 |
| 7. | | Were you limited in pursuing your hobbies or other leisure time activities? | 1 | | 2 | | 3 | 4 |
| 8. | | Were you short of breath? | 1 | | 2 | | 3 | 4 |
| 9. | | Have you had pain? | 1 | | 2 | | 3 | 4 |
| 10. | | Did you need to rest? | 1 | | 2 | | 3 | 4 |
| 11. | | Have you had trouble sleeping? | 1 | | 2 | | 3 | 4 |
| 12. | | Have you felt weak? | 1 | | 2 | | 3 | 4 |
| 13 | | Have you lacked appetite? | 1 | | 2 | | 3 | 4 |
| 14. | | Have you felt nauseated? | 1 | | 2 | | 3 | 4 |
| 15. | | Have you vomited? | 1 | | 2 | | 3 | 4 |
| 16. | | Have you been constipated? | 1 | | 2 | | 3 | 4 |
|  | |  |  | |  | |  |  |
|  | | **During the past week** | **Not**  **at all** | | **A**  **little** | | **Quiet**  **a bit** | **Very**  **much** |
| 17. | | Have you had diarrhea? | 1 | | 2 | | 3 | 4 |
| 18. | | Were you tired? | 1 | | 2 | | 3 | 4 |
| 19. | | Did pain interfere with your daily activities? | 1 | | 2 | | 3 | 4 |
| 20. | | Have you had difficulty in concentrating on things, like reading a newspaper or watching television? | 1 | | 2 | | 3 | 4 |
| 21. | | Did you feel tense? | 1 | | 2 | | 3 | 4 |
| 22. | | Did you worry? | 1 | | 2 | | 3 | 4 |
| 23. | | Did you feel irritable? | 1 | | 2 | | 3 | 4 |
| 24. | | Did you feel depressed? | 1 | | 2 | | 3 | 4 |
| 25. | | Have you had difficulty remembering things? | 1 | | 2 | | 3 | 4 |
| 26. | | Has your physical condition or medical treatment interfered with your family life? | 1 | | 2 | | 3 | 4 |
| 27. | | Has your physical condition or medical treatment interfered with your social activities? | 1 | | 2 | | 3 | 4 |
| 28. | | Has your physical condition or medical treatment caused you financial difficulties? | 1 | | 2 | | 3 | 4 |
|  | |  |  | |  | |  |  |

**For the following questions please circle the number between 1 and 7 that best applies to you**

| 29. | How would you rate your overall health during the past week? | |
| --- | --- | --- |
| \| 1 \| 2 \| 3 \| 4 \| 5 \| 6 \| 7 \| \| --- \| --- \| --- \| --- \| --- \| --- \| --- \| \| Very poor \|  \|  \|  \|  \|  \| Excellent \| | |  |
| 30. | How would you rate your overall quality of life during the past week? | |
| \| 1 \| 2 \| 3 \| 4 \| 5 \| 6 \| 7 \| \| --- \| --- \| --- \| --- \| --- \| --- \| --- \| \| Very poor \|  \|  \|  \|  \|  \| Excellent \| | |  |

© Copyright 1995 EORTC Quality of Life Group. All rights reserved. Version 3.0

**Supplementary Table S3. Details of severe adverse related to mobilization treatment**

|  | **All patients** | **CG** | **G** | **p-value** |
| --- | --- | --- | --- | --- |
| All patients | 136 (100%) | 68 (100%) | 68 (100%) | 1.00 |
| Patients with adverse events CTCAE 1 |  |  |  |  |
| Infection and Infestations | 1 (1%) | 1 (1.5%) | 0 (0%) | 1.00 |
| Patients with adverse events CTCAE 2 |  |  |  |  |
| Infections and infestations* | 3 (2%) | 2 (3%) | 1 (1%) | 1.00 |
| Cardiac disorder | 1 (1%) | 1 (1%) | 0 (0%) | 1.00 |
| Patients with adverse events CTCAE 3 |  |  |  |  |
| Infections and infestations | 2 (1%) | 1 (1%) | 1 (1%) | 1.00 |
| Gastrointestinal disorder | 2 (1%) | 1 (1%) | 1 (1%) | 1.00 |
| Eye disorder | 1 (1%) | 0 (0%) | 1 (1%) | 1.00 |
| Psychiatric disorder | 1 (1%) | 0 (0%) | 1 (1%) | 1.00 |
| Vascular disorder | 1 (1%) | 1 (1%) | 0 (0%) | 1.00 |
| Nervous system disorder | 1 (1%) | 0 (0%) | 1 (1%) | 1.00 |
| Metabolism and nutrition disorder | 1 (1%) | 0 (0%) | 1 (1%) | 1.00 |
| General disorder** | 1 (1%) | 1 (1%) | 0 (0%) | 1.00 |

Data are n (%). CG=chemotherapy plus G-CSF group. G=G-CSF only group. *1 patient hat 2 different infections. **Multi organ failure.

**Supplementary Table S4. Quality of Life Assessment (EORTC Q30)**

|  | **CG** | **G** | **p-value** |
| --- | --- | --- | --- |
| Patients | 66 (97%) | 60 (88%) |  |
| Difference (t1-t2) in QoL-Score^1^ | 0 (-5,5) | -1 (-12,4) | 0.14 |
| Difference (t1-t2) in perceived health status^2^ | 0 (-1,0) | 0 (-1,1) | 0.17 |
| Difference (t1-t2) in perceived QoL^3^ | 0 (-1,0) | 0 (0,1) | 0.09 |

Data are n (%) or median (IQR). t1-t2= Difference between first and second timepoint of survey. QoL=quality of life. ^1^median score of questions 1-28 of EORTC QLQ-C30 Version 3.0. ^2^Question 29 of EORTC QLQ-C30 Version 3.0. ^3^Question 30 of EORTC QLQ-C30 Version 3.0.

**Supplementary Figure S1. Numeric pain rating scale**

**Pain Scale:** Please mark the number between 0 (no pain) and 10 (worst pain imaginable) that best describes your experience.


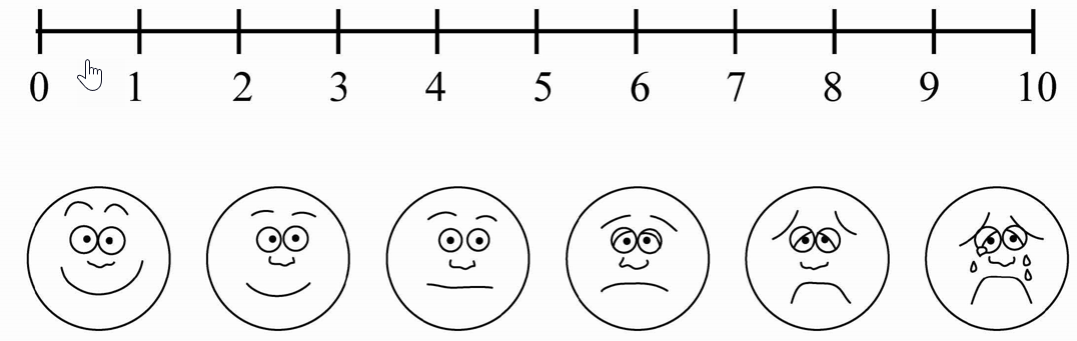

Supplement: Supplementary file 1 — Supplementary tables and figures [file 41409_2024_2468_MOESM1_ESM.docx]
